# An integrated model of the human cornea as a linear biaxial birefringent medium

**DOI:** 10.1038/s41598-024-55800-4

**Published:** 2024-03-01

**Authors:** Marcelina Sobczak, Agnieszka Jóźwik, Piotr Kurzynowski

**Affiliations:** 1 Department of Optics and Photonics, Wroclaw University of Science and Technology, Wybrzeże Wyspiańskiego 27, 50-370 Wrocław, Poland; 2 School of Optometry, Indiana University, 800 Atwater Ave, Bloomington, IN 47405 USA

**Keywords:** Biomaterials, Medical research

## Abstract

A novel model of human corneal birefringence is presented. The cornea is treated as a homogeneous biaxial linear birefringent medium in which the values of the binormal axes angle and organization of the main refractive indices vary continuously from the apex to the limbus. In its central part, the angle between binormal axes is 35°, and para centrally, it smoothly increases to 83.7°. The values of the main refractive indices (*n*_x_, *n*_y_, *n*_z_) change, as well as their order, from *n*_x_ < *n*_z_ < *n*_y_ to *n*_z_ < *n*_x_ < *n*_y_. The transition between these two states was described with a normal distribution (μ = 0.45, σ = 0.1). The presented model corresponds with the experimental results presented in the literature. To our knowledge, it is the first model that presents the anisotropic properties’ distributions of the entire cornea. The presented model facilitates a better understanding of the corneal birefringence phenomenon directly related to its lamellar structure.

## Introduction

The cornea is a transparent shell that, together with the sclera, forms the outer surface of an eyeball. Corneal transparency is guaranteed by its characteristically organized structure. The cornea comprises six layers: epithelium, Bowman’s membrane, stroma, Dua’s layer, Descemet’s membrane, and endothelium, where the stroma is the layer largely responsible for the optical corneal properties. The stroma consists of interlaced lamellae that are 10 to 200 µm wide and 0.2 to 2.5 µm thick^1,2^. Each lamella is composed of organized collagen fibers (fibrils) with diameters smaller than the wavelength of light and as well as ground substance. The building material of fibrils is collagen, mainly type I—an intensely positively birefringent medium with respect to fibrils’ length^3^. Together with ground substance, they determine the intrinsic birefringence of the lamellae. The fibrils in each lamellae run parallel to the corneal surface, except where they branch out to interact with fibrils in adjacent lamellae^2,4^. The mutual angle between successive lamellar layers is random close to the corneal apex. Outside the corneal apex, most of the lamellae start to demonstrate a preferred orientation in the inferior–superior (sagittal plane) and nasal–temporal (transverse plane) directions^5,6^. In contrast to the optically important central cornea, in the peripheral cornea, one can observe additional circumferential limbal lamellae. The arrangement of the corneal lamellae and their structure help maintain the overall shape of the tissue, determine the mechanical properties of the stroma, and are also responsible for optical properties, including corneal clarity and birefringence.

Many models of the lamellar orientation have been developed^6–15^. Boote et al. proposed a model which, combines all the pre-existing models. They used microfocus wide-angle X-ray scattering to quantify the relative proportion and orientation of collagen fibrils across the human corneolimbal interface at 50 µm intervals^12^. In their model, the lamellae are orthogonal to each other (mostly in horizontal and vertical orientations) in the central and para central corneal areas. In the peripheral region of the cornea, these fibers bend and connect to the tangential fibrils that form a reinforced limbal ring. The additional lamellar strands, originating from the sclera, appear and cross the perilimbal area obliquely. On the other hand, based on in vivo study, Misson proposed the spatial model of the elliptical or hyperbolic orientation of the lamellae in the stroma^13,14^. The model assumed the existence of two foci with the highest lamellar density on opposite sides in the limbal area. The arrangement of the stromal lamellae and its optical parameters (e.g., collagen’s intrinsic birefringence) cause the cornea to be considered a linear birefringent medium.

Observation of the human cornea in white light using a linear crossed polariscope reveals dark cross-like isogyres, which are lines of constant polarization direction. Additionally, colorful concentric isochromes, representing lines of equal phase difference, emerge on the cornea’s periphery. This finding aligns with the observations made on inorganic, optically birefringent substances. The first study on corneal birefringence were made at the turn of the nineteenth century^16,17^, but interest increased in the second half of the twentieth century. The studies showed that some corneas behave like curved, optically negative biaxial crystal plates^17–23^ while others like uniaxial ones^23–27^.

The first measurements using polariscopic methods were performed on feline corneas^24,25,28^. It was shown that the corneal behavior could be caused by a random distribution of stacked lamellar layers. In 1975, Wang et al.^26^ compared human corneas with those of other species (e.g., pig, cow, rabbit). They demonstrated that these corneas can be treated as a curved birefringent crystal and that their optical path difference changes from 0 at the corneal apex and increases toward the limbus. They also stated that the lowest birefringence was observed in human corneal samples. According to the human corneal study by Bour and Lopes Cardozo, the corneal phase difference increases more along diagonal corneal cross-sections than along the horizontal and vertical ones^29^. In addition, the local crystal orientation axis is of a radial-type in all tested species^24–26,29^.

Most of the researchers confirmed that the human cornea could be described as a linear birefringent medium^14,18,21,23,27,30–38^. The phase difference (retardation) differs between measured corneas but the trend stays the same. It is minimal in the center with an increase towards the limbus^18,20,21,27,30,32–38^. The rate of change in the paracentral and peripheral region is relatively small^23^. The azimuth angle distribution is radial-type and becomes constant in the peripheral area^23,39,40^. The effect of dichroism and light depolarization is mostly negligible^31,41^. Apart from the ellipticity of the corneal birefringence, the existence of a slow and fast axis is also an issue to be considered. Most researchers state that the cornea is a biaxial medium^8,18,19,21,23,27,31,39^. Blokland and Verhelst, based on their in vivo measurements described cornea in the central area (approximately 6 mm) as a linear biaxial birefringent crystal with a fast axis perpendicular to its surface and a slow axis along the corneal surface in the inferonasal direction^18^. The angle between binormal axes is 35° and its maximum birefringence equals 1.59 × 10^−3^. Bone and Draper examined the central area of the cornea in their in vitro experiments^21^. They found that this area behaves like a negative biaxial birefringent crystal. The angle between the binormal axes was 12–40°, and the tilt angle of the optical axis plane was between 135° and 179° for the right corneas and between 1° and 45° for the left ones, which is the inferonasal direction. The retardation was less than 200 nm. Fanjul-Velez et al.^33,34,38^ conducted a series of both in vivo and in vitro studies and concluded that the cornea in the center can be described as a linear biaxial birefringent crystal, and the distribution in the periphery is quasi-radial with high birefringence. Several studies also revealed other properties of the anisotropic parameters of the cornea. With increasing pupil size, measured retardation also increased, while ocular birefringence remained linear and azimuthal angle changed without a clear trend^30^. The value of the phase difference is lowest at the corneal vertex and increases radially towards the limbus. The increase in the phase difference value is also observed as the stromal depth increases^32^. Furthermore, an enantiomorphism was observed between the two eyes of each subject^23,27,42^. The birefringence distribution showed a mirror symmetry between the left and right eyes in each of the measured subjects.

Knighton et al.^22^ measured the corneal birefringence of the central and paracentral areas of the cornea (approximately 8 mm in diameter) and found that the properties of the cornea can be described using 3 distinct models. The first two described the corneal tissue as a biaxial linear birefringent material placed between two spherical surfaces with a fast axis perpendicular to the corneal plane. In the first model, the slow axis is directed in the inferonasal direction, whereas in the second, it is oriented horizontally. The third model assumes that the cornea can be treated as a uniaxial linearly birefringent crystal with the optical axis perpendicular to its plane. The authors found that the corneal birefringence varies significantly between humans and also varies in value within a single cornea. Mastropasqua et al.^42^ due to technical factors measured only para-pupillary area of the cornea. They noticed that human corneas can be described by two models: the linear birefringent uniaxial one and biaxial ones. They did not rule out that the uniaxial model is actually biaxial (pseudo-one-axis crystal), but the angle between the normal axes is very small and they were not able to define it. Therefore, it can be assumed that the cornea is generally biaxial, but the angle in this model is very small and falls outside the measurement area. The differences in the models presented above are gathered in Supplementary Table S1.

The aim of our work is to find a single integrated model of the cornea with regard to its optical properties consistent with the experimental results cited above. The cornea, and especially the stroma, is a multilayer system that exhibits birefringent properties. The purpose of the numerical simulations was to find a single uniform model describing the cornea’s optical properties from the optical axis to the limbus consistent with its birefringence measured experimentally. The cornea will be presented as one anisotropic optical layer with locally variable properties, such as thickness, eigen axis orientation, and birefringence.

## Methods

### Birefringent media

In birefringent media, the refractive index $n$ depends on the direction of light propagation and the light polarization state. Therefore, light propagation must be considered in three dimensions. The unit vector of the direction of the wave propagation $\hat{s}$ in birefringent media is given by Eq. (1):

1 $$\hat{s} = \left( {s_{x} ,s_{y} ,s_{z} } \right),$$

where $s_{x}$, $s_{y}$, $s_{z}$ are scalar components expressed in Cartesian notation.

In anisotropic media, the relation between the electric displacement field and the electric field is not proportional as in an isotropic media and must be described using the permittivity tensor $\overline{\varepsilon }$ (Eq. (2)) where the $n_{x,y,z}$ are the directional main indices of refraction:

2 $$\overline{\varepsilon } = \left| {\begin{array}{*{20}c} {\varepsilon_{x} } & 0 & 0 \\ 0 & {\varepsilon_{y} } & 0 \\ 0 & 0 & {\varepsilon_{z} } \\ \end{array} } \right| = \left| {\begin{array}{*{20}c} {n_{x}^{2} } & 0 & 0 \\ 0 & {n_{y}^{2} } & 0 \\ 0 & 0 & {n_{z}^{2} } \\ \end{array} } \right|.$$

Using Maxwell’s equations and the mentioned relation, a biquadratic equation for refractive index $n$ of a wave results (Eq. 3):

3 $$n^{4} - pn^{2} + q = 0,$$

where

4 $$p = \frac{{s_{x}^{2} \varepsilon_{x} \left( {\varepsilon_{y} + \varepsilon_{z} } \right) + s_{y}^{2} \varepsilon_{y} \left( {\varepsilon_{x} + \varepsilon_{z} } \right) + s_{z}^{2} \varepsilon_{z} \left( {\varepsilon_{x} + \varepsilon_{y} } \right)}}{{s_{x}^{2} \varepsilon_{x} + s_{y}^{2} \varepsilon_{y} + s_{z}^{2} \varepsilon_{z} }}$$

and

5 $$q = \frac{{\varepsilon_{x} \varepsilon_{y} \varepsilon_{z} }}{{s_{x}^{2} \varepsilon_{x} + s_{y}^{2} \varepsilon_{y} + s_{z}^{2} \varepsilon_{z} }}.$$

This biquadratic equation has two positive solutions—here it is $n_{1,2}$. This means that two waves can propagate at different velocities in a given direction of light propagation $\widehat{s}$. Consequently, different refractive indices occur. The unit vector of the wave propagation $\widehat{s}$ and the permittivity tensor $\overline{\varepsilon }$ determine the coefficients $p$, $q$ of Eq. (3). Hence the solutions of this equation ${n}_{\mathrm{1,2}}={n}_{\mathrm{1,2}}\left(\widehat{s};\overline{\varepsilon }\right)$ also depend on these two variables, as well as the birefringence $\Delta n$, defined as the difference in the refractive indices ${n}_{\mathrm{1,2}}$ of propagated waves.

Whether the considered medium is optically active or inactive (the medium is elliptically or linear birefringent, respectively), the relationship between the refractive indices can be different. If the main refractive indices ${n}_{x}$, ${n}_{y}$, and ${n}_{z}$ differ from each other, the medium is biaxial. In this medium, there are two light propagation directions (binormal axes) along which birefringence equals zero, and the angle between them is called a binormal angle ($\beta$). When two of the three refractive indices are the same, the medium is uniaxial, and $\beta =0^\circ /180^\circ$ dependently on the sign of the medium.

**Figure 1:**
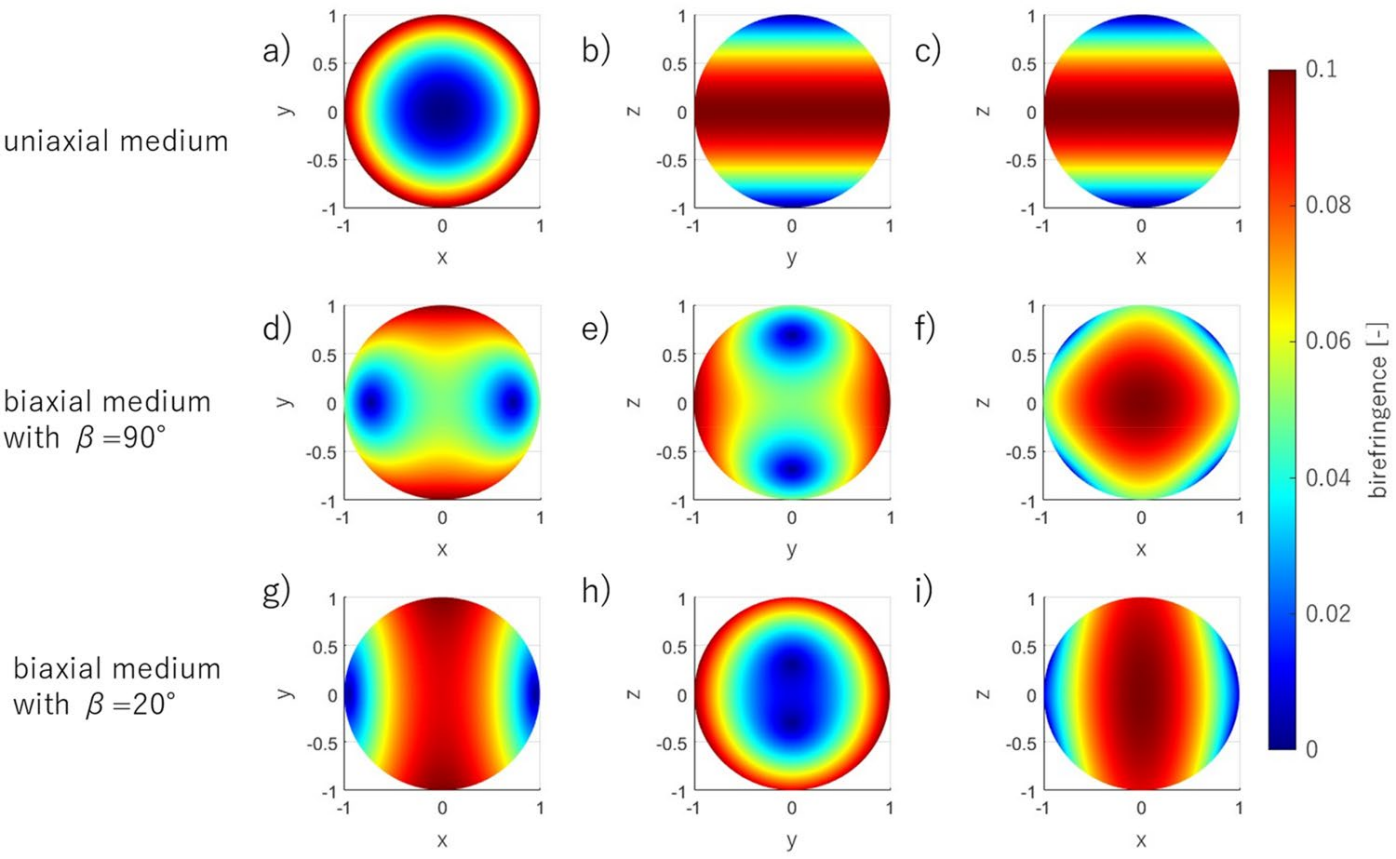
The birefringence distribution on the unit sphere seen on the X–Y (**a**, **d**, **g**), Y–Z (**b**, **e**, **h**), and X–Z (**c**, **f**, **i**) planes for an uniaxial medium with refractive indices ${n}_{x}={n}_{y}=1.5$, ${n}_{z}=1.6$ (**a**–**c**), a biaxial medium with angle between binormal axes β = 90° and refractive indices ${n}_{x}=1.5$, ${n}_{y}=1.55$, ${n}_{z}=1.6$ (**d**–**f**), and a biaxial medium with β = 20° and refractive indices ${n}_{x}=1.5$, ${n}_{y}=1.59$, ${n}_{z}=1.6$ (**g**–**i**).

The birefringence is calculated at each of the designated points along the normal vector to the surface of the unit sphere. The unit sphere was used as a first step of the corneal shape modeling to show the behavior of the anisotropic media other than a flat parallel plate. In the Cartesian axes, the refractive indices are ${n}_{x}$, ${n}_{y}$, and ${n}_{z}$, respectively. The birefringence $\Delta n$ depends on the unit vector $\widehat{s}$ and source position, thus the measured birefringence distribution, but also the shape of the conoscopic figures can vary with different observation directions. An example of results is presented in Fig. 1. The birefringence is calculated at each of the designated points along the normal vector to the surface. The birefringence distributions are shown on the sphere that corresponds qualitatively to the corneal surface assuming the point light source is placed in the origin of the coordinate system. Figure 1a–c show the uniaxial medium with ${n}_{x}={n}_{y}<{n}_{z}$, and Fig. 1d–i the biaxial medium with ${n}_{x}<{n}_{y}<{n}_{z}$. In Fig. 1d–f relation of refractive indices is as follows: ${n}_{y}-{n}_{x}={n}_{z}-{n}_{y}$, whereas in Fig. 1g–i is ${n}_{y}-{n}_{x}\gg {n}_{z}-{n}_{y}$. The varying relation between the local refractive indices not only causes different shapes of the conoscopic figures but also affects the angular orientation between the binormal axes in the biaxial media (for medium in Fig. 1d–f and g–i the angles are 90° and 20°, respectively).

### Distributions of anisotropic parameters in experiments

**Figure 2:**
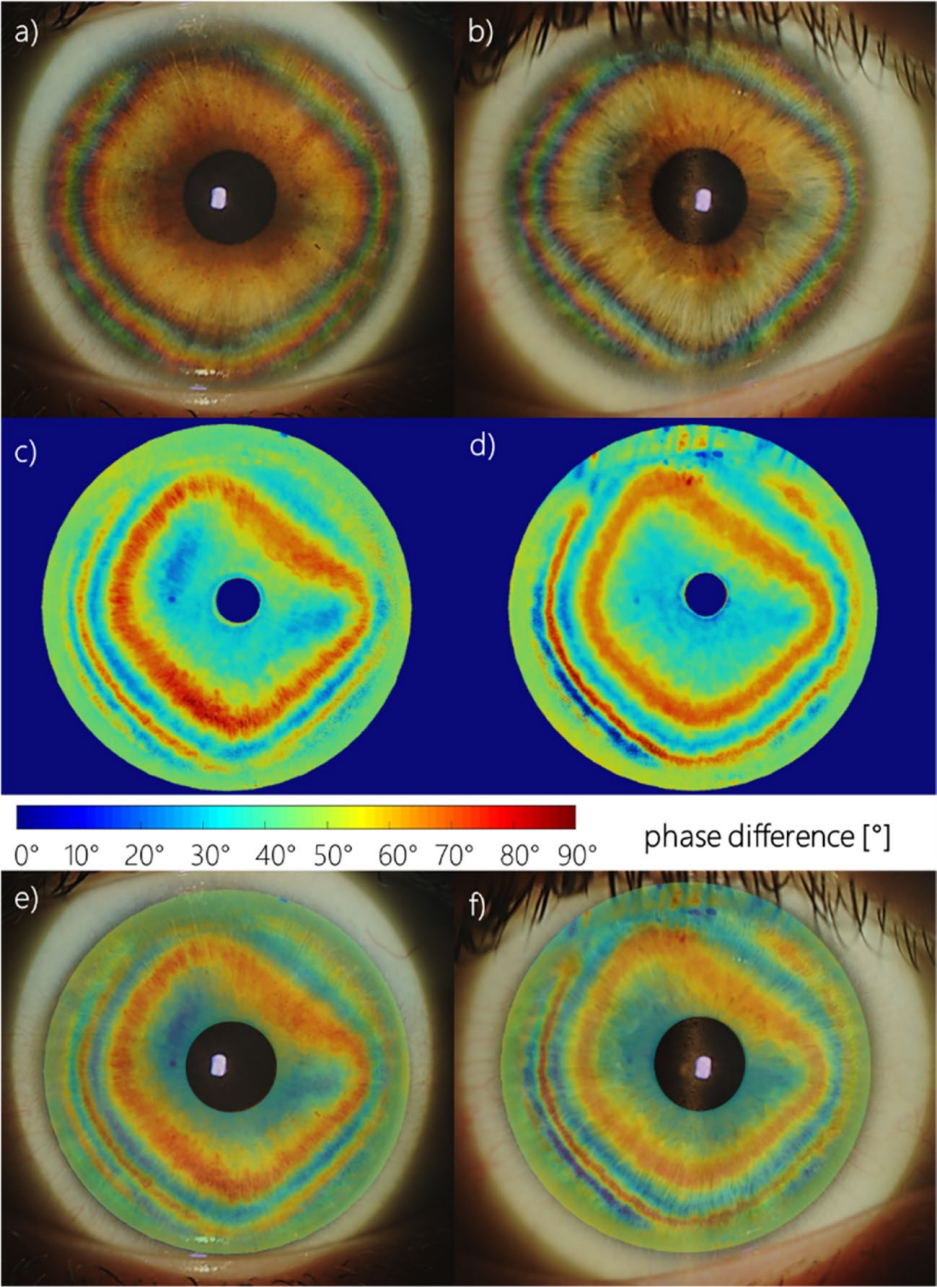
The example of the results acquired from the experiment for two different right eyes; conoscopic figures (**a**, **b**), phase difference maps (**c**, **d**), conoscopic figures and phase difference distributions superimposed on each other (**e**, **f**).

The experimental results were obtained using two optical systems described in detail in Sobczak et al.^39,40,43^ The studies were approved by the Ethics Committee of Wroclaw Medical University (KB 329/2014) and adhered to the tenets of the Declaration of Helsinki. Informed consent was obtained from all participants after they had been fully informed of their requirements and about the purpose and procedures of the project. Concisely, the first system consists of a circular polarizer fitted to a slit lamp working in white light^43^. The circular polarizer is placed in front of an eye, so the light passes through it when entering the eye and again as the light leaves the eye. Thus, it polarizes light coming into the eye and acts as an analyzer for light returning from the eye. This system enables us to directly image the conoscopic figures that demonstrate the anisotropic nature of the cornea (Fig. 2a,b). The colorful fringes appear due to a dispersion phenomenon. The phase difference is wavelength-dependent; hence, its values vary depending on the spectral distribution of the light source. The second optical system is a partial Mueller matrix polarimeter working in reflective mode^39,40^. The crucial part of this system is a polarization state generator (PSG) working as a polarizer and analyzer placed directly before the eye. PSG comprises a linear polarizer and two liquid crystal retarders. This allows us to produce and analyze the light (λ = 660 nm) of six polarization states (four linear and two circular). The phase difference and azimuth angle can be computed with the use of a Mueller formalism and Stokes vector (Fig. 2c,d). This formalism results in solutions with a limited range for the azimuth angle [0°, 45°), and for phase difference [0°, 90°). The birefringence distribution was calculated along the nasal-temporal line dividing the retardation (phase difference expressed in nanometers) by the corneal thickness acquired in the same cross-section. By overlapping the results obtained from both methods (Fig. 2e,f), it is evident that they are in good qualitative agreement and provide data to be accounted for by our model.

**Figure 3:**
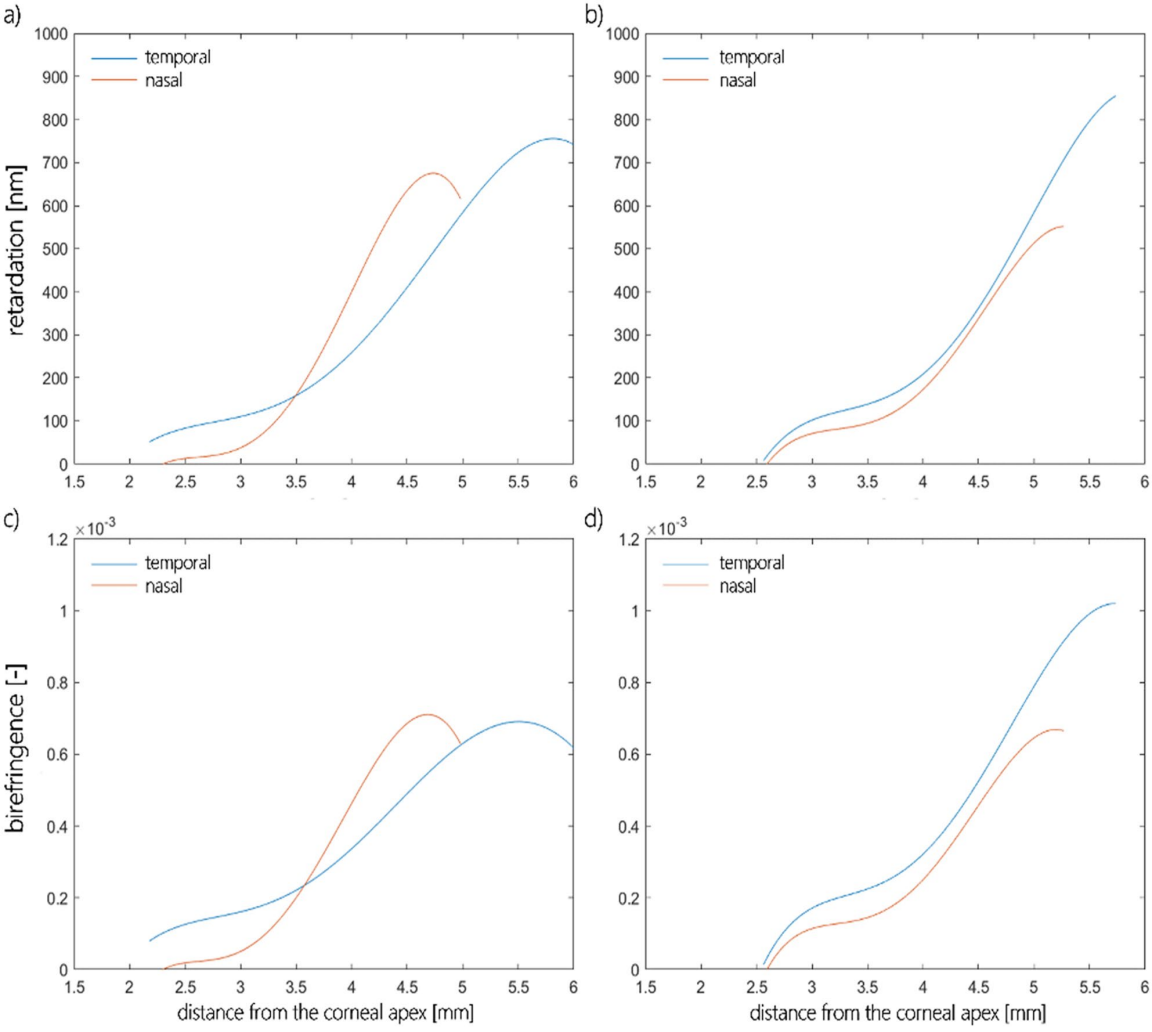
Retardation (**a**, **b**), birefringence distribution (**c**, **d**) for horizontal cross-section divided into nasal (orange line) and temporal parts (blue line) of two eyes (first—**a**, **c**; second—**b**, **d**) obtained in the experiment.

Examples of the retardation and birefringence distributions along nasal-temporal cross-section are shown in Fig. 3. They both have minimal values close to the central part of the cornea and maximal values at the limbus. On average, retardation and birefringence reach their highest values at approximately 800 nm and 1.0 × 10^−3^, respectively. The angle between the binormal axes is calculated to be ~ 82.9° in the peripheral area (~ 4.0 mm from the corneal apex). It should be emphasized that the most extreme values of the anisotropic parameters vary between eyes. These changes may also appear within a single subject between the left and right eyes. These distributions show pronounced differences between their nasal and temporal parts. Although noteworthy, this nasal-temporal disparity is not considered in the presented model.

### Model assumptions

The corneal stroma is a multilayer medium that exhibits birefringent properties. For numerical simulations giving an integrated description of its optical properties, it can be presented as one anisotropic optical layer with locally variable properties, such as thickness, eigen axis orientation, and birefringence. The modeled cornea would have to fulfill the following assumptions.

**Figure 4:**
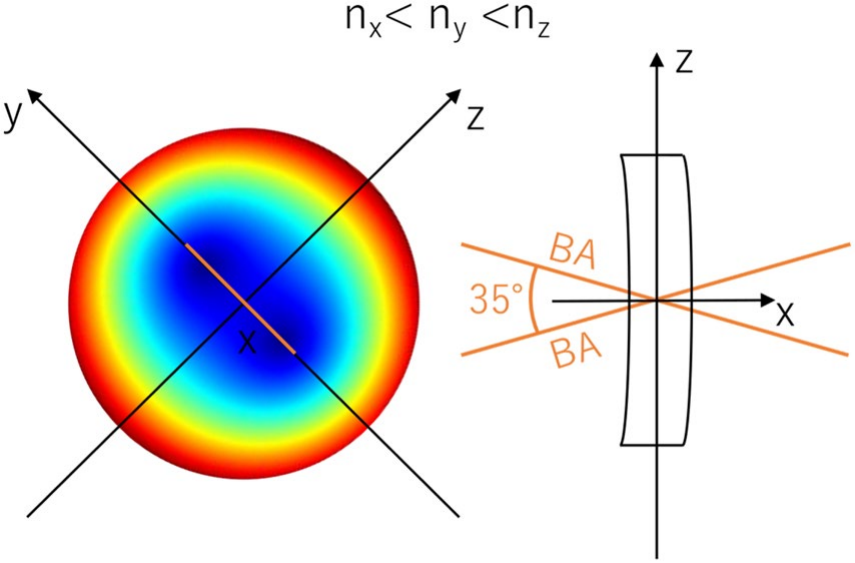
Orientation of the local principal axes x, y, and z and the angle between the binormal optical axes BA (orange lines) for the central part of the cornea presented on its entire surface^29^.

**Figure 5:**
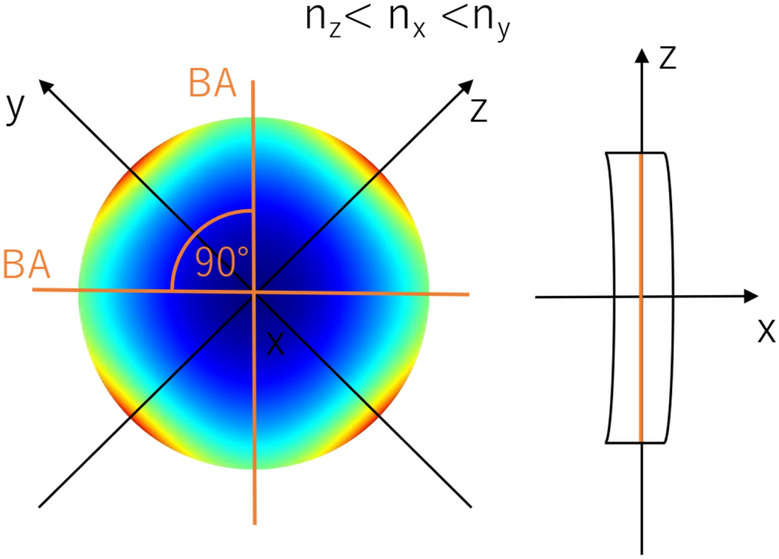
Orientation of the local principal axes x, y, and z and the angle between the binormal optical axes BA (orange lines), assuming that the entire cornea is described by the variables of its para central part.

- The shape of the front and the rear corneal surfaces should be aspheric. The corneal radius of the anterior surface, R_1_ = 7.76 mm with conic constant k_1_ = − 0.10 and the corneal radius of the posterior surface, R_2_ = 6.52 mm with k_2_ = − 0.30 were taken from the Goncharov model of the eyeball^44^.
- The model assumes that the cornea is rotationally symmetrical. Therefore, the corneal apex is in its center, and the curvature and thickness are the same in the nasal and temporal parts of the cornea.
- The corneal thickness change with radius from the corneal axis results from the characteristics of the first and second corneal surfaces, and the model assumes a central corneal thickness of 0.55 mm^44^.
- Although the corneal refractive index varies slightly between corneal layers^45^, in this model, we treat the cornea as a single layer element with a constant mean refractive index of n = 1.3777^46^.
- The corneal diameter is set to 12 mm, although the average corneal diameter is 11.72 ± 0.42 mm (range for males 11.04–12.50 mm, for females 10.70–12.58 mm)^47^. We used the notation of the corneal zones presented in Supplementary Fig. S1.
- The birefringence distributions and the angle between the binormal axes ($\beta$) must be roughly the same as those measured experimentally in in vivo experiments^18,39,40^. The distribution of the birefringence and phase difference (retardation) must grow from the paracentral to the peripheral corneal area, while in the central corneal distributions they reach their minimum values.
- A realistic lighting model must be adopted. The point light source should be placed in such a position as to enable the generation of conoscopic figures consistent with the experiments and the transmission of light through the cornea, which implies solving the problem of light returning from the optical system of the eye to the observer. The issue of reflection/scattering appears regarding the iris and the retina. The amount of light reflected from the iris/retina participating in the formation of conoscopic figures varies depending on the observed corneal location.
- The light propagates along X-axis perpendicular to the corneal surface and centrally this overlaps with the optical axis of the eye.
- The modeling process adopts the wavelength λ = 660 nm from the experiment to closely mimic the cited experimental conditions.
- The cornea, based on the experimental data, can be treated as a biaxial birefringent medium with the binormal axes located in the superior-temporal/inferior-nasal plane. Two of the three local principal axes lie in a plane tangent to the respective surfaces of the cornea, and the third axis is perpendicular to this plane, i.e., perpendicular to the surface of the cornea.
- In the central corneal, the angle between binormal axes (β) is about 35° (Fig. 4)^14,18^. The X-axis refers to the lowest refractive index, while the Y-axis to the highest one, and ${n}_{y}-{n}_{z}<{n}_{z}-{n}_{x}$.
- The isochrome experimental results for the peripheral cornea showed quadrangular distributions (Fig. 2), distinctly different from the concentric circle-shaped isochromes in the central area (as shown in Fig. 4). It is necessary to locally change the distribution of the main refractive indices of the equivalent birefringent medium and/or their order along these axes (Fig. 5). The lowest and highest refractive indices run along the Y- and Z-axes, respectively. Furthermore, the numerical relationships between the main refractive indices are ${n}_{x}-{n}_{y}={n}_{z}-{n}_{x}$, from which it can be determined that the angle between binormal axes is β = 90° and lies in the corneal plane. In theory, it provides quadrangle-shaped isochromes (Fig. 1f). The shape of the isochromes acquired during the experiments is not perfectly square, indicating that the angle between binormal axes is slightly different from 90°.

### Building the model

The model was built with the following conditions: corneal curvature and thickness, distribution of refractive indices along the local principal axes, and the distance of a point light source from the cornea.

**Figure 6:**
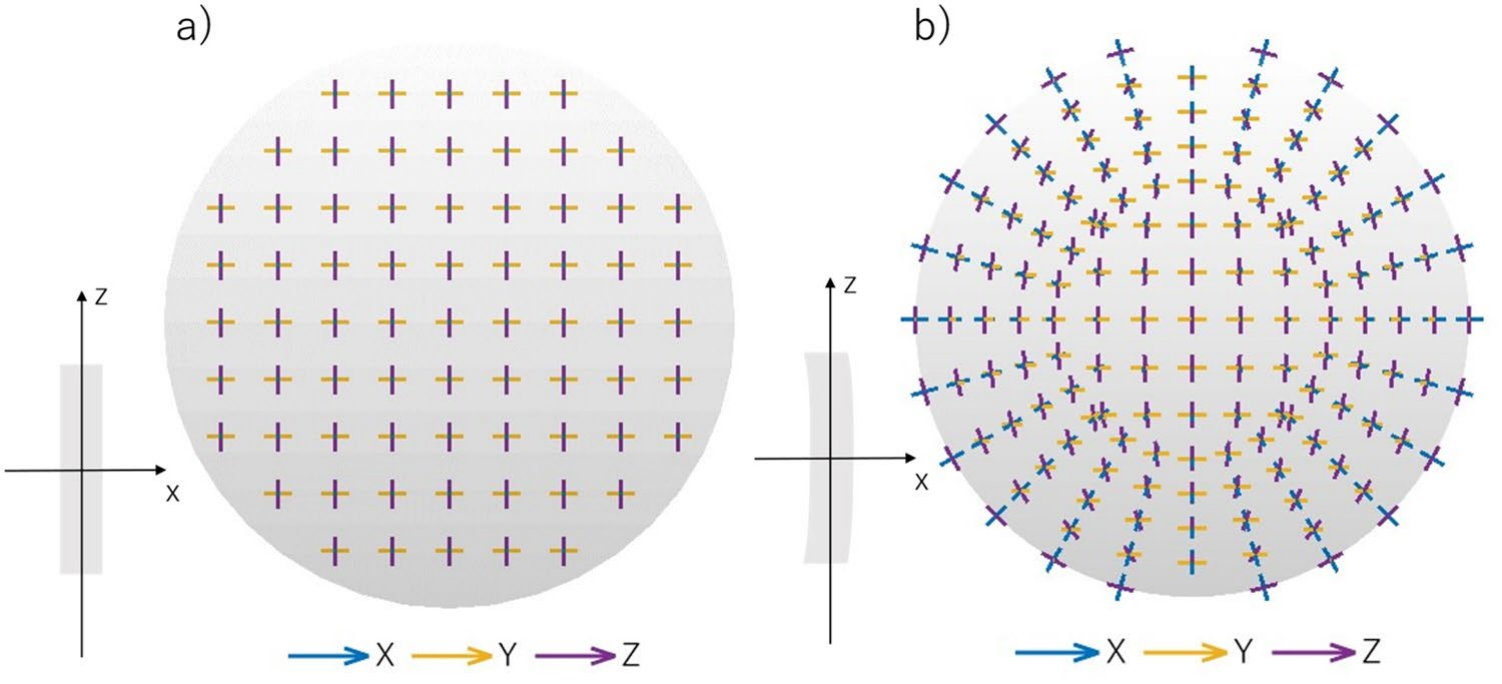
The orientation of the local axes on the flat disc (**a**), and on the cornea-shaped surface (**b**) projected onto a plane. With this curvature change, two local axes (Y, Z) stay locally parallel to the surface while the third remains perpendicular to it. The consequence of this is what appears to be a radial distribution when projected onto a plane. Both discs’ diameter of 12 mm corresponds to the diameter of the cornea.

The cornea was initially treated as a flat disc with a constant thickness and a diameter of 12 mm corresponding to the diameter of the cornea. The assumption is that at each point of the flat disc’s birefringent medium, the orientation of the local principal axes is such that two of these axes lie in the plane of the disc, while, the third is perpendicular to its surface (Fig. 6a). It does not mean that the birefringent properties are constant across the cornea, as they are also determined by the three main refractive indices ${n}_{x},{n}_{y}$ and ${n}_{z}$. These will change from the center of the cornea to the periphery of the cornea as discussed below.

Next, the flat disc was curved and its thickness varied using the corneal parameters taken from the Goncharov eye model^44^, described in the model assumptions section. The cornea is described by two aspherical surfaces that are rotationally symmetrical around the eye’s optic axis. At each point of the created surface, the local distribution of the eigen axes of the birefringent medium remains Cartesian (transverse axes have a horizontal-vertical orientation everywhere However, when projected onto a two-dimensional registration plane (a Y–Z plane), as for example the image plane of a camera, the organization of eigen axes changes due to the curvature of cornea (Fig. 6b).In the center of the cornea, the eigen axes are still seen as organized in a Cartesian way, whereas, in peripheral region, they appear to be arranged in a radial way. The consequence of such observation is that the azimuth angle distribution appears to be radial, especially in the peripheral region. This is consistent with the experimental observations of this parameter.

The way conoscopic figures are formed in the optical system is an equally important concern in modeling corneal birefringence. Its crucial element is the rays’ path from the source to the registration plane (the Y–Z plane). During the modeling process, two approaches were utilized. The first assumed that the point light source was placed in front of the cornea at a certain distance. The beam encounters the outer air-corneal interface and double refracts as it passes through the cornea then exits through the cornea’s inner surface at the cornea-aqueous interface. The second refraction angle is much smaller (in this model it has been neglected) due to the minor difference in refractive indices between the cornea and the aqueous humor (corneal n = 1.377, aqueous humor n = 1.336). Next, a substantial part of the light reflects from the iris surface, and of that which passed through the pupil a small proportion reflects from the retina. It was assumed that the iris could be treated as a plane mirror. After the light passes through the cornea again, it produces the conoscopic figures in the registration plane. As most of the light is reflected from the iris and very little from the retina, the reflection from the retina was neglected in the modeling process to make the ray tracing process less complex. To make certain the assumption that the iris was acting as a plane mirror was not critical to our results, a second approach was also implemented. The light source was placed in the anterior chamber as a secondary source at the iris plane. In this model, light first goes through the inner corneal surface (without refraction), then splits into two rays, faster and slower, passes through the cornea-air interface and passes into the air. The following reasoning was implemented for both approaches.

**Figure 7:**
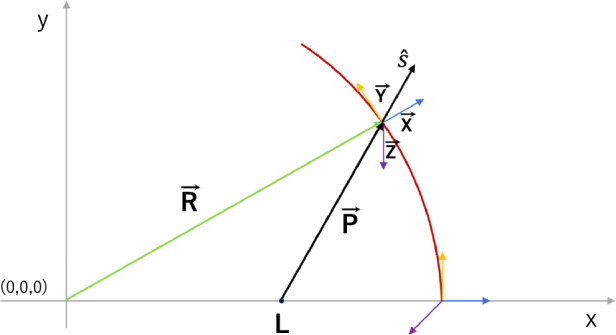
Ray tracing in the modeled cornea (red line); $\overrightarrow{R}$—a vector defining the curvature of one of the corneal surfaces (green arrow), $\overrightarrow{P}$—a vector determining the light path (black arrow), *L*—a light source, $\overrightarrow{X}$, $\overrightarrow{Y}$, $\overrightarrow{Z}$—local principal axes changing their arrangements with corneal curvature' changes (blue, yellow, and purple arrows, respectively), $\widehat{s}$—a unit vector of the wave propagation direction (the components of the unit vector are distributed along the local principal axes).

The cornea consists of two aspherical surfaces with different radii of curvature and conic constants (described in detail in the model assumptions section). On each surface, the eigen axes of the cornea remain orthogonal to the plane at every point (orange axes in Fig. 7). The rays, represented as a vector $\overrightarrow{P}$, from the point light source model (whose starting point is on the main X-axis) encounter one of the corneal surfaces at various angles that do not overlap with any of the eigen axes in most cases (Fig. 7).

Due to the above, it is necessary to calculate the scalar components ${s}_{i}$ (relating to the local X, Y, and Z axes) of the unit vector of the wave propagation direction $\hat{s}$ (Eq. 1) in birefringent media (Eq. 6):

6 $$s_{i} = \frac{{\vec{P} \circ \vec{i}}}{{\left| {\vec{P}} \right|}}; i = x, y, z,$$

where $\circ$ is a dot product.

The difference of the optical paths *R* (retardation) resulting from the corneal birefringence is an integral of the slowly variable birefringence function $\Delta n$ with respect to the geometrical path $s$ when light passes through two points (*A* and *B*) of the cornea (Eq. 7):

7 $$R = \mathop \smallint \limits_{A}^{B} \Delta n \cdot ds.$$

The birefringence at inner and outer surfaces of the cornea ($\Delta n_{{\text{A}}} ,\Delta n_{{\text{B}}}$) are generated as a difference in refractive indices (solutions of the quadratic equation (Eq. (3)). Assuming that the birefringence is approximated with a linear function of the optical path (associated with the corneal thickness along the light ray), $\Delta n_{{\text{A}}}$ and $\Delta n_{{\text{B}}}$ differ. Therefore, for a given light ray propagating through the cornea, the mean value $\Delta n$ of the birefringence calculated on the first and second surfaces of the cornea is calculated (Eq. 8):

8 $$\Delta n = \frac{{\Delta n_{{\text{A}}} + \Delta n_{{\text{B}}} }}{2}.$$

This implies that the total retardation can be calculated as a product of the mean birefringence $\Delta n$ and the geometric path length $d$ for light passing through the cornea (Eq. 9):

9 $$R = \Delta n \cdot d.$$

Having the optical path difference *R* and knowing the wavelength of the light used in the experiments (λ = 660 nm), it is also possible to determine the phase difference.

In a transition from the corneal center to the limbus, the order of the main refractive indices must change continuously from ${n}_{x}<{n}_{y}<{n}_{z}$ to ${n}_{z}<{n}_{x}<{n}_{y}$ (Table 1). However, the mean refractive index should be constant and equal to 1.3777. As it was assumed, the human cornea is described by a biaxial medium, in which the angle between binormal axes (β) in its central area is equal to 35° and rises to 83.7° in the periphery. The disparity from the 90° that would provide the square-shape isochromes is based on the isochromes’ shape observation (deviates from squareness) and the experimental results.

**Table 1:** The order of refractive indices in central (column 2) and peripheral (column 3) parts of the cornea along local axes.

Refractive indices	Central part	Peripheral part
*n*_x_	1.3764	1.3770
*n*_y_	1.3776	1.3776
*n*_z_	1.3775	1.3765

**Figure 8:**
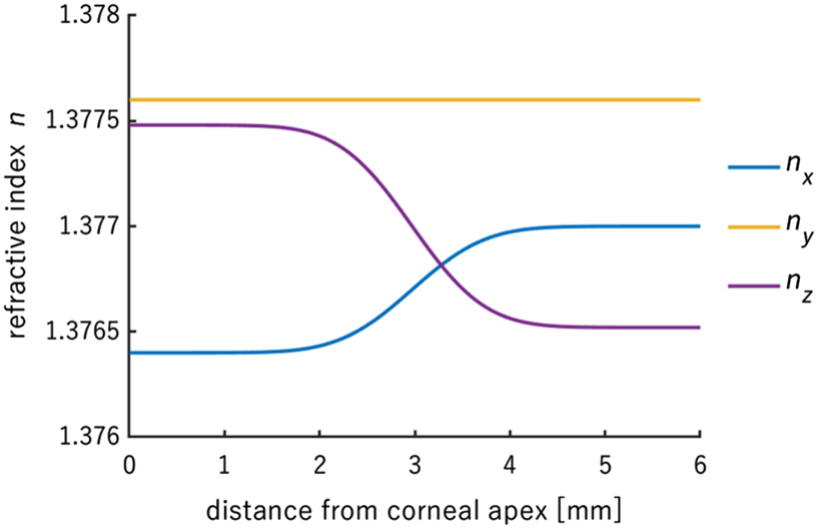
Refractive indices distributions ($n_{x} ,\,n_{y} , \,n_{z}$) along principal axes (*x*, *y*, *z*) from the corneal apex to the peripheral area. It is assumed that the corneal is rotationally symmetrical.

The resultant corneal retardation is the combination of the retardation generated in the central and peripheral parts. To achieve this, the fluent change of refractive indices was modeled according to the normal distribution *P* (μ = 0.45, σ = 0.1) and was used as follows (Eq. 10):

10 $${n}_{i}=P\cdot{n}_{i, central}+\left(1-P\right)\cdot {n}_{i,peripheral}, i=x,y,z,$$

where ${n}_{i, central}$ are the refractive indices along the $i=x,y$, and $z$ local principal axes of central part, and ${n}_{i, peripheral}$ for the peripheral part, respectively. The results are presented in Fig. 8.

## Results

The computation was performed in a Matlab® environment (MathWorks, version R2022b). The retardation distributions were generated separately for the central and peripheral corneal parts. The resultant distribution and horizontal cross-sections of retardation and birefringence were also made for points over the entire corneal surface. The variable parameters in the calculations were the distance of the point light source *L* from the cornea, combinations of refractive indices along the local eigenvalues of the equivalent birefringent medium and the angle between the binormal axes.

### Localization of the light source

Two approaches to light source localization were taken. In the first approach, the point light source was placed in front of the outer corneal surface at a distance of 650 mm. Comparing that distance with the corneal radii, we can posit that the propagated wave is planar. The second approach treated the light scattered from the iris surface as a secondary light source. That light source was in the anterior chamber at a distance of 3.5 mm deep to the outer corneal surface, that is, in the iridial plane. Both approaches to light localization yielded similar results. As the outside light source better approximates the experimental environment, we decided to only present the results for this particular approach.

### Retardation and birefringence distributions

**Figure 9:**
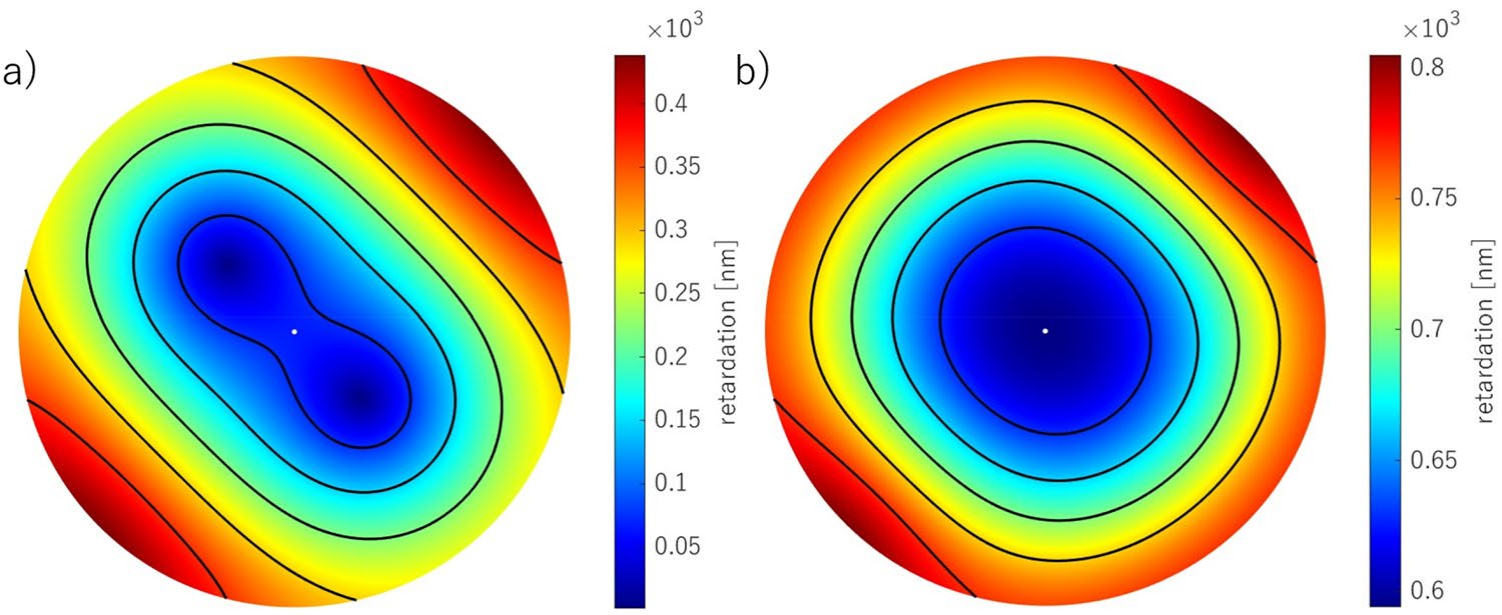
The component retardation distributions for the refractive indices of the central (**a**) and peripheral (**b**) corneal areas mapped on its whole surface; corneal diameter equals 12 mm.

The retardation was simulated based on the assumption presented in methods that the corneal birefringent properties change from the corneal apex to the limbus. In the central area, the cornea is described as a biaxial medium, and its order of the main refractive indices is as follows: ${n}_{x}<{n}_{y}<{n}_{z}$, and the angle between the binormal axes is 35° (Fig. 9a) ^18^. In the paracentral and peripheral areas, the cornea stays biaxial, but the angle is increased to 83.7° (Fig. 9b). Moreover, the order of the main refractive indices changed to ${n}_{z}<{n}_{x}<{n}_{y}$. At an early stage of the model, the angle in the peripheral area was stated as 90° because, in theory, it provides quadrangle-shaped isochromes. The change in this assumption provided isochromes more similar to the experimental results. A possible reason for this change in angle, is that it is due to the location of the attachments of the extraocular muscles. The lines connecting the medial rectus to the lateral rectus and similarly the superior to the inferior rectus are not orthogonal, but they intersect at an angle smaller than 90°. Next, based on the Tillaux spiral, the distances of the extraocular muscles’ attachments to the corneal apex are distinct, so the tension acting on the cornea is different in these different specific directions. This may indicate that the cornea experiences a stress birefringence accounting for or contributing to observed shifts in the axes of birefringence from 90 degrees. Moreover, the angle between lines connecting inflection points of the first isochrome might be calculated from the parameters presented in Sobczak et al., and the mean is equal to 82.9° in the adult cohort^40,43^.

**Figure 10:**
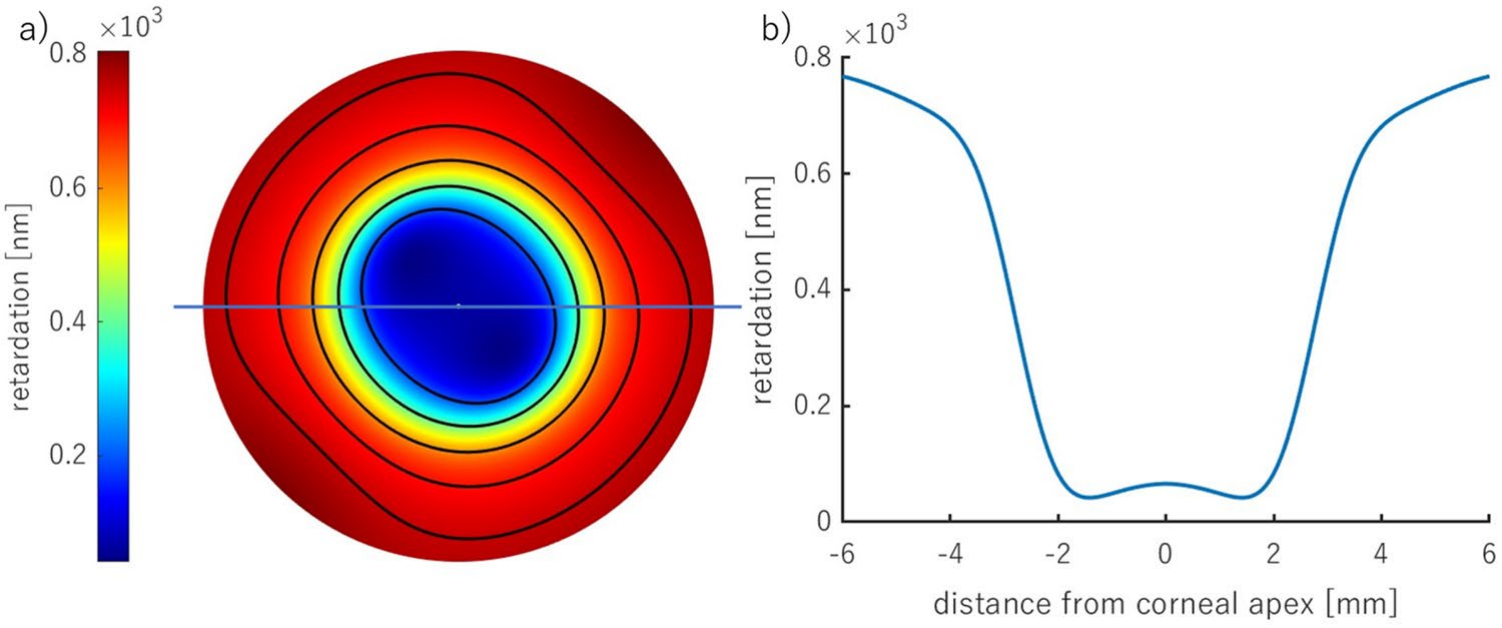
The resultant retardation distribution resulting from the refractive indices' changes described by the normal distribution (corneal diameter of 12 mm) (**a**), the horizontal cross-section (marked as a blue line in (**a**)) of the corneal retardation (**b**).

The resultant retardation distribution was obtained with the combination of the central and off-central distributions using the normal distribution function (μ = 0.45, σ = 0.1) on the refractive indices (Fig. 8). The result is presented in Fig. 10 a. This proportion of the distribution causes the distance between two local minima of this distribution to be located at about 4.0 mm. It corresponds with the optimal pupil size in the sense of minimizing the eye's aberrations and diffraction correction. The retardation values, in particular corneal areas, correspond to those measured in the experiments (Fig. 2c,d). The horizontal cross-section of the retardation (Fig. 10b) has a similar distribution to the one obtained from the experiment (Fig. 3a,b).

**Figure 11:**
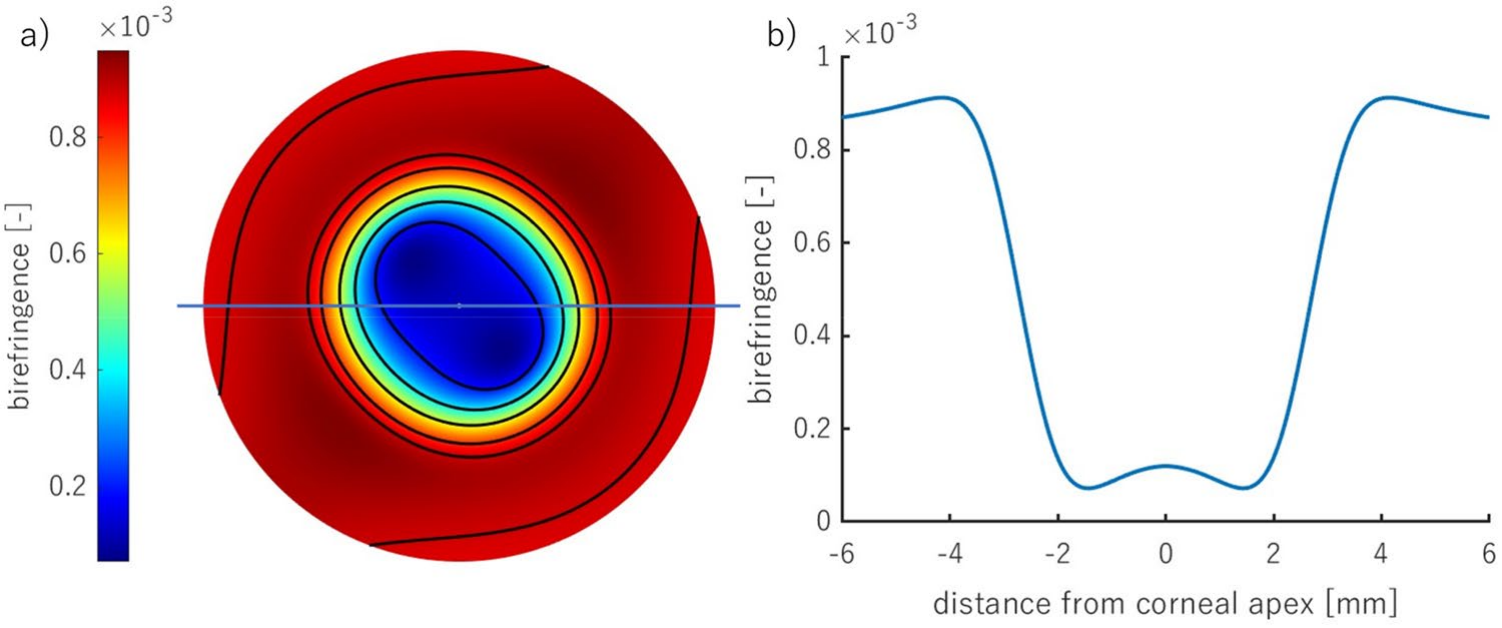
The resultant distribution of the modeled corneal birefringence (corneal diameter of 12 mm) (**a**), the horizontal cross-section (marked as a blue line in (**a**)) of the corneal birefringence (**b**).

The observation of the cornea in a circular polariscope (Fig. 2a,b) or the measurements of the anisotropic properties (Fig. 2c,d) acquire, at the first stage, the isochromes (lines of equal phase difference). That is why the above results were shown based on the retardation distribution. However, the retardation and birefringence distributions have different arrangements due to varying corneal thickness (Fig. 11). The birefringence increases from the apex towards the periphery obtaining its maximum value  ~ 4 mm from the center, then its values decrease. These results are also consistent with the experimental data (Fig. 3c,d).

### Azimuth angle distribution

The azimuth angle distribution observed in the experiments has a radial distribution. It is in line with the model assumption about the local principal axes (Fig. 6b). The model states that two of the three axes are parallel to the corneal surface, and the last is orthogonal, which can be interpreted in the projection onto the two-dimensional plane as a radial distribution. This geometrical effect follows from the curvature of the corneal surfaces. If the cornea were a flat shell, the principal axes across the entire cornea would be seen as Cartesian.

## Conclusions

A novel integrated model of corneal birefringence was proposed. It treats the cornea as a biaxial linear birefringent medium, with optical properties that smoothly vary from the apex to the limbus. The central part is a medium with an angle between binormal axes equal to 35°, whereas, in the peripheral part – 83.7°. The presented results fit the experimental data well, which can be observed in the birefringence and retardation cross-sectional distributions (Figs. 10b, 11b). The retardation distributions have quasi-quadrangular shapes in the peripheral area both in the proposed model and in the presented experimental results. These same qualitative outcomes were shown in the literature^9,11,13,18,27,32,42^. Two of the local principal axes are parallel to the corneal surface at every point, and the third axis is orthogonal. In the plane, it can be perceived as radial, which is consistent with the azimuth angle distribution.

Limitations of this model include that it does not incorporate the light reflected from the retina, under the assumption that its contribution to image formation is small and may be neglected. Next, this model considers the iris as a flat mirror reflecting the incoming light. However, the structure of the iris is much more complicated, and the diffuse scattering from its surface was not taken into account in the modeling process though the second model, treating a source in the iris plane produced similar results to the model treating the iris as a mirror. The corneal birefringence could have components not only of form and intrinsic birefringence, but also of stress birefringence caused by extraocular muscle tension. The stress birefringence would not be expected to be radially symmetric and would be expected to vary with eye position as the extraocular muscle tensions change. Our model does not attempt to treat these additional complexities.

The presented model facilitates a better understanding of the corneal birefringence phenomenon directly related to its lamellar structure. Early detection of the structural changes caused by corneal ectasias such as keratoconus, can help to prevent their progression^48–50^. In the specific case of keratoconus, corneal cross-linking has stabilized corneal stromal structure preventing progression to penetrating keratoplasty^51,52^. Retardation distribution can be easily and inexpensively observed with a circular polariscope attached to a slit lamp; a commonly used optical instrument in ophthalmic and optometric practice. Imaging and geometrical quantification of corneal isochromes is a potentially valuable tool for early detection of clinical pathology affecting the cornea though it is not yet common in clinical practice. Integration and interpretation of clinical data through a corneal model can allow a deeper understanding of both normal corneal optical properties as well as the properties of pathological corneas. The development of a corneal model for this purpose constitutes fundamental research in this area of study.

### Supplementary Information

Supplementary Information.

## Data Availability

Data underlying the results presented in this paper are not publicly available at this time but may be obtained from the corresponding author—Marcelina Sobczak, upon reasonable request.
