# Supplementary material for: An integrated model of the human cornea as a linear biaxial birefringent medium
Source: Sci Rep. 2024 Mar 1;14:5077. doi: 10.1038/s41598-024-55800-4 (PMC10907592; doi:10.1038/s41598-024-55800-4)
Supplement: Supplementary file 1 — Supplementary Information. [file 41598_2024_55800_MOESM1_ESM.docx]

**Table S1. The review of the selected studies on the corneal birefringence**

| Authors | Method | Area of cornea | | Subject | Linear birefringence | in vivo | ex vivo |
| --- | --- | --- | --- | --- | --- | --- | --- |
|  |  | central | para central |  |  |  |  |
| Valentin (1861)^17^ | cross-polariscope | x | x | human | biaxial |  | x |
| Stanworth et al. (1950)^24^, Naylor (1953)^25,28^ | polariscope | x | x | cat | uniaxial |  | x |
| Wang et al. (1975)^26^ | polariscope | x | x | human, pig, cow, rabbit | uniaxial |  | x |
| Bour et al. (1981)^29,53^ | polariscope | x | x | human | uniaxial | x |  |
| Blokland et al. (1987)^18^ | ellipsometry using Mueller matrix | x | x | human | biaxial | x |  |
| Bueno (2000)^30^ | polarimetry | x | x | human | uniaxial | x |  |
| Bueno et al. (2001)^31^ | polarimetry using Mueller matrix |  |  | human | uniaxial |  | x |
| Bueno et al. (2002)^23^ | liquid-crystal imaging polariscope |  | x | human | uniaxial | x |  |
| Knighton et al. (2002)^19^ | Purkinje images polarimetry | x |  | human | biaxial | x |  |
| Jaroński et al. (2003)^20^ | polarimetry using Jones matrix | x | x | human | biaxial |  | x |
| Götzinger et al. (2004)^37^ | phased-resolved PS-OCT | x | x | human |  | x |  |
| Hitzenberger et al. (2006)^34^ | PS-OCT | x | x | human |  |  |  |
| Bone et al. (2007)^21^ | polarizing microscope | x |  | human | biaxial | x |  |
| Misson (2007, 2010)^13,14^ | circular polarization biomicroscopy |  |  | human | biaxial | x |  |
| Knighton et al. (2008)^22^ | scanning laser polarimetry | x |  | human | uniaxial, biaxial | x |  |
| Fanjul-Velez et al. (2009, 2010)^33,34,38^ | PS-OCT | x | x | human | biaxial | x |  |
| Bueno (2011)^37^ | double-pass polarimetry | x | 8 mm | human | biaxial para central | x |  |
| Mastropasqua et al. (2017)^42^ | polarimetric interferometry |  | x | human | biaxial paracentral, uniaxial limbal | x |  |
| Beer et al. (2017)^27^ | PS-OCT | x | x | human | uniaxial | x |  |
| Sobczak et al. (2021)^39,40,44^ | double-pass Mueller type polarimeter |  | x | human | biaxial | x |  |

PS-OCT – polarization sensitive optical coherence tomography; para central – outside the central area of the cornea


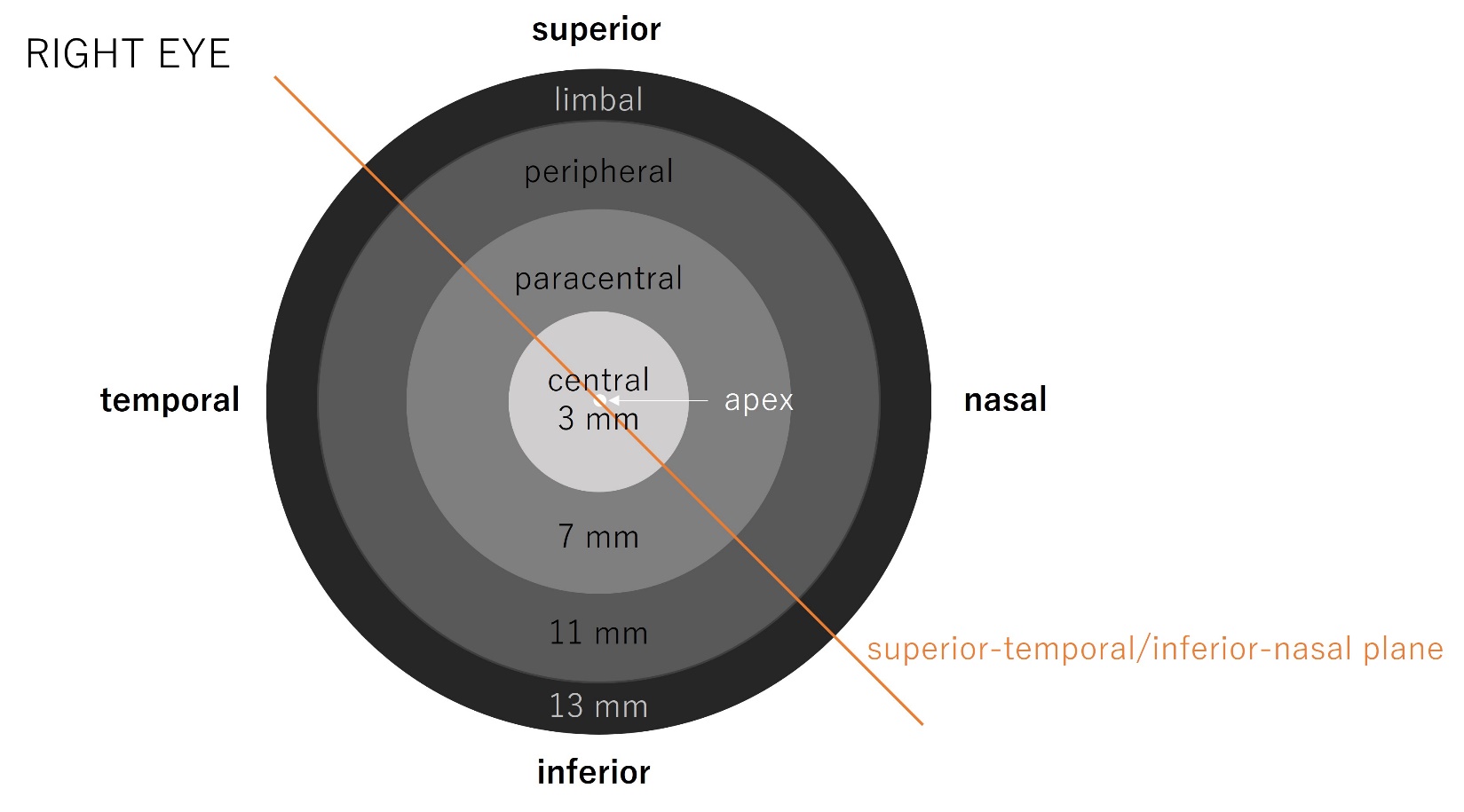


Fig. S1. The corneal zones (apex, central, paracentral, peripheral, limbal) in the right eye with the main directions (superior, inferior, nasal, temporal) and the superior-temporal/inferior-nasal plane (orange line).
